# Dissecting the Role of BET Bromodomain Proteins BRD2 and BRD4 in Human NK Cell Function

**DOI:** 10.3389/fimmu.2021.626255

**Published:** 2021-02-26

**Authors:** Adam P. Cribbs, Panagis Filippakopoulos, Martin Philpott, Graham Wells, Henry Penn, Henrik Oerum, Viia Valge-Archer, Marc Feldmann, Udo Oppermann

**Affiliations:** ^1^ Botnar Research Center, Nuffield Department of Orthopedics, Rheumatology and Musculoskeletal Sciences, National Institute of Health Research Oxford Biomedical Research Unit (BRU), University of Oxford, Oxford, United Kingdom; ^2^ Structural Genomics Consortium, University of Oxford, Oxford, United Kingdom; ^3^ Arthritis Centre, Northwick Park Hospital, Harrow, United Kingdom; ^4^ Roche Innovation Center Copenhagen A/S, Hørsholm, Denmark; ^5^ Bioscience, Research and Early Development, Oncology R&D, AstraZeneca, Cambridge, United Kingdom; ^6^ Kennedy Institute of Rheumatology Nuffield Department of Orthopedics, Rheumatology and Musculoskeletal Sciences, Botnar Research Centre, Oxford, United Kingdom; ^7^ Freiburg Institute of Advanced Studies, Freiburg, Germany; ^8^ Oxford Centre for Translational Myeloma Research, Oxford, United Kingdom

**Keywords:** BET bromodomain, BRD2, BRD4, NK cell, epigenetics (DNA methylation histone modifications)

## Abstract

Natural killer (NK) cells are innate lymphocytes that play a pivotal role in the immune surveillance and elimination of transformed or virally infected cells. Using a chemo-genetic approach, we identify BET bromodomain containing proteins BRD2 and BRD4 as central regulators of NK cell functions, including direct cytokine secretion, NK cell contact-dependent inflammatory cytokine secretion from monocytes as well as NK cell cytolytic functions. We show that both BRD2 and BRD4 control inflammatory cytokine production in NK cells isolated from healthy volunteers and from rheumatoid arthritis patients. In contrast, knockdown of BRD4 but not of BRD2 impairs NK cell cytolytic responses, suggesting BRD4 as critical regulator of NK cell mediated tumor cell elimination. This is supported by pharmacological targeting where the first-generation pan-BET bromodomain inhibitor JQ1(+) displays anti-inflammatory effects and inhibit tumor cell eradication, while the novel bivalent BET bromodomain inhibitor AZD5153, which shows differential activity towards BET family members, does not. Given the important role of both cytokine-mediated inflammatory microenvironment and cytolytic NK cell activities in immune-oncology therapies, our findings present a compelling argument for further clinical investigation.

## Introduction

Natural killer cells are cytolytic lymphocytes belonging to the innate immune system and are involved in anti-viral and anti-tumor responses (1) and are recognized as major players in immune-mediated anti-tumor therapies (2). Their function is regulated by the activation of a number of activating and inhibitory receptors that bind to specific ligands expressed on the surface of target cells. In particular, NK cells mediate their cytolytic function through the engagement of activating receptors, such as NKG2D, DNAM-1, NKp30, NKp46, and NKp44 (3, 4), or following pro-inflammatory cytokine stimulation (5). Following activation, NK cells mediate killing of target cells through two major pathways that require direct contact between NK cells and their target cells (6). The first mechanism involves killing mediated by cytotoxic molecules (*e.g.* perforin and granzymes) that are stored in secretory granules of lysosomal origin (7). The second pathway involves the engagement of death receptors with their ligands (*e.g.* Fas/FasL) that results in caspase-dependent apoptosis. Moreover, NK cells are poised to release cytokines such as IFN-**γ**, TNF-α and growth factors that can initiate inflammatory responses mediated by both the innate and the adaptive arm of the immune system.

**Figure 1:**
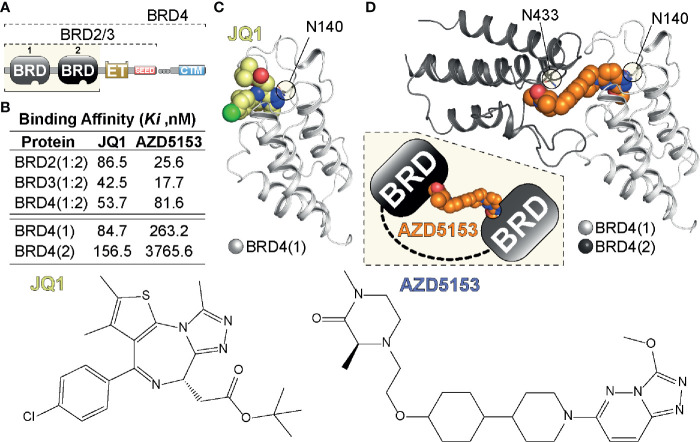
Tandem BET BRD targeting. **(A)** Domain organization of BET (BRD2,3,4) proteins highlighting the N-terminal tandem bromodomain (BRD) modules, the Extra-Terminal (ET) and C-terminal motif (CTM) as well as SEED region. **(B)** Binding affinities of single (JQ1) and tandem (AZD5153) BET inhibitors previously determined in a Fluorescent polarization (FP) assay (11). **(C)** Single BRD4 site engagement by JQ1. The inhibitor is shown as a CPK model engaging BRD4(1) *via* Asn140 (N140), the amino acid residue in the acetyl-Lys binding pocket of BRD4(1) (12). **(D)** Model of tandem BRD4(1:2) engagement by AZD5153. The inhibitor (shown as a CPK model) engages BRD4(1) *via* N140 and BRD4(2) *via* N433 linking the tandem BRD modules as indicated in the inset. The model was generated from PDBs 5KHM and 2OUO (13).

Bromodomains are proteins that contain modules of ~110 amino acids that recognize and bind acetylated lysine residues in histones and other proteins. Recognition of acetylated chromatin marks by BRDs enables the regulation of gene expression through a wide range of activities. BRDs can act as scaffolds that enable the recruitment of large protein complexes or they can act as transcription factors themselves. Additionally, BRDs contain several catalytic domains that enable them to act as methyltransferases, ATP-dependent re-modellers or histone acetyltransferases and helicases (8) Bromodomain and extra-terminal domain (BET) proteins are a family of transcriptional mediators that regulate gene expression (8, 9). The BET family consists of BRD2, BRD3, BRD4, and BRDT which bind histone acetylated lysine residues *via* two highly conserved amino-terminal bromodomains, BD1 and BD2 found in each family member (10) (**Figure 1**). A number of BET bromodomain inhibitors have been developed to understand the utility of BETs in oncology, with each having different specificities for BD1 and BD2 (12, 14, 15). However, no BET bromodomain inhibitor can reliably distinguish between different BET family members (16).

Despite extensive work undertaken to explore the function and mechanisms regulating BET bromodomains in myeloid and T cells (10, 17), little is known about the role of BET bromodomains in NK cells. Previous work has shown that BET inhibitors reduce the expression of IFN-**γ** in stimulated NK cells, suggesting a potential role for BET BRDs in NK cell function (18). However, the precise mechanism of how BETs regulate NK cell function has not been explored. In the present study, we aim to evaluate the role and importance of BET BRDs in regulating NK cell cytolytic and inflammatory function. Our study clearly demonstrates the central role BET BRDs play in regulating NK cell cytolytic and inflammatory function. Furthermore, our work suggests that targeted strategies for inhibiting different BET family members may in the future be an effective therapeutic strategy for both autoimmunity and cancer.

## Materials and Methods

### Reagents

IL-15 (R&D systems), IL-2(PeproTech), Pam3CSK (InvivoGen), MCSF (PeproTech), GMCSF (PeproTech). CD3/28 activation beads were purchased from Invitrogen. JQ1(+) and JQ1(−) were used at a concentration of 1 µM, while AZD5153 was used at a concentration of 0.1 µM for all experiments, unless otherwise stated. Antibodies for flow cytometry were purchased from BioLegend. Media and sera were tested for endotoxin before being used in experiments.

### Cell Isolation and Cell Culture Experiments

NK cells were isolated from either venous bloody obtained from healthy volunteers, or from platelet pheresis residues obtained from the Oxford National Blood Transfusion Service. Peripheral blood was obtained from Rheumatoid Arthritis patients attending the early RA clinic at Northwick Park Hospital, London. The study was approved by the London Riverside Research Ethics Committee (REC) 07/H0706/81 and the Oxford Research Ethics Committee 06/Q1606/139. All samples were obtained ethically, and the material was used in accordance with the terms of the informed consent. All patients were diagnosed according to the American College of Rheumatology (ACR) Eular 2010 criteria.

Human peripheral blood mononuclear cells were isolated by Ficol density gradient centrifugation, and CD56+ cells were isolated using the Dynabeads® UntouchedTM Human NK cell kit (Invitrogen), as per manufacturer’s instructions. Isolated NK cells were cultured in IMDM (Gibco) supplemented with 5% heat inactivated fetal calf serum.

Monocytes were isolated using the Pan Monocyte Isolation kit (Miltenyi) and were cultured in the presence of MCSF (10 ng/ml) and GMCSF (10 ng/ml) for 24 h before being stimulated with Pam3CSK (30 ng/ml). CD4+ T cells were isolated using Dynabeads® Untouched™ Human CD4 kit (Invitrogen) and cultured in the presence of IL-2 (50 ng/ml) for 24 h before being stimulated with CD3/28 activation beads (1:1 ratio, Invitrogen), as per manufacturer’s instructions. CD8+ T cells were isolated using Dynabeads® Untouched™ Human CD8 kit (Invitrogen) and then cultured in the presence of IL-2 for 24 h before being stimulated with CD3/28 activation beads (Invitrogen), as per manufacturer’s instructions.

### IC50 ELISA Experiments

ELISA (eBioscience) was used to measure the concentration of IFN-γ cytokines within the cell culture supernatant, which was performed according to manufacturer’s conditions. All standards and samples were measured in triplicate.

### Flow Cytometry

Cells were analyzed using a BD LSR Fortessa™ flow cytometer following staining with fluorochrome conjugated antibodies. Prior to staining, cells were fixed using the fix buffer set (Biolegend), according to the manufacturer’s instructions. Antibodies used within this study were conjugated anti-CD3 (UCHT1, Biolegend), anti-CD56 (N901, Beckman Coulter), anti-NKp30 (P30-15, Biolegend), anti-NKp44 (P44-8.1, BD), anti-NKp40 (29A1.4, Biolegend), anti-TNF-α (Mab11, eBioscience), anti-IFN-**γ** (485.B3, Biolegend). Annexin V/PI staining was performed according to the manufacturer’s protocol (Biolegend). The data was analyzed using FlowJo 10.7.1 software.

### Quantitative RT-PCR

Total RNA was extracted using TRIzol reagent (Invitrogen) and a Direct-zol RNA miniprep kit (Zymo Research). Complimentary DNA was generated using a SuperScript II RT kit (Invitrogen), according to the manufacturer’s instructions. Reverse transcription PCR using specific primers was used to determine gene expression. Gene expression levels were normalized relative to the expression of *β*-actin. All samples were measured in triplicate. Specific primers used within this study are:

**Table:** 

Gene	Forward primer	Reverse primer
IFNG	TCGGTAACTGACTTGAATGTCCA	TCGCTTCCCTGTTTTAGCTGC
TNF	CCTCTCTCTAATCAGCCCTCTG	GAGGACCTGGGAGTAGATGAG
NCR1	TGGACCCGAAGTGATCTCG	TCCTTGAGCAGTAAGAACATGC
NCR2	GGCTCTCAGGCACAATCCAAG	GCTGAAGCCTCCTTACACCA
NCR3	CCCCTGAGATTCGTACCCTG	CTCCACTCTGCACACGTAGAT

### Locked Nucleic-Acid Knockdown Experiments

Knockdown experiments were performed using locked nucleic-acids (LNA). LNAs are nucleic acids with a methylene bridge connecting the 2′ oxygen and 4′ carbon of the ribose ring. This results in increased stability and target affinity, which results in efficient knockdown without the requirement for transfection reagents or transduction techniques (19). Our oligonucleotides were designed and synthesized by Santaris A/S (now Roche Innovation Center Copenhagen) and purified by HPLC, desalted using a Milliprep membrane and verified by LC-MS. NK cells were cultured at a concentration of 1 × 106 cells/ml and stimulated with IL-15 (10ng/mL). LNAs were added to the culture medium at a concentration of 5 µM and then cultured for 7 days before beginning experiments.

### NK Cell Killing Experiments

NK cells killing experiments were performed as previously described (20). Briefly, 1 × 106 NK cells were stimulated with IL-15 in the presence of DMSO, JQ1(+) or AZD5153 for 24 h. The cells were washed twice in fresh medium before being added to the NK cell killing assay. In a 96-well round bottom plate 1 × 105 NK cells at an effector-target ratio of 5:1 for 4 h. Target cells were JURKAT (clone E601, ATCC) and K562 (clone CCL-234, ATCC). Lactate dehydrogenase release was measured in 50 µl of supernatant using the Cytotox 96 assay kit (Promega), according to manufacturer’s instructions.

In parallel, NK cell degranulation assay was performed as previously described (21). Briefly, pre-treated NK cells were cultured with target cells at a 1:5 ratio in the presence of anti-CD107a-Fitc antibody (H4A3, Biolegend) and incubated for 1 h at 37°C. Protein transport inhibitor (eBiosciences) was added for the final 3 h of culture. After staining with anti-CD56, Anexin V and PI, the sample was assessed by flow cytometry on a BD LSR Fortessa™ instrument. For blocking antibody studies anti-IgG1 (12G8G11, BioLegend), anti-NKp30 (P30-15, BioLegend), NKp-44 (P44-8.1, BD), and NKp-46 (29A1.4, BioLegend) were added at the beginning of the assay. Data analysis was performed using the FlowJo program.

### RNA Isolation and Sequencing Library Preparation

Total RNA was extracted using TRIzol reagent (Invitrogen) and a Direct-zol RNA miniprep kit (Zymo Research) according to the manufacturer’s protocol. The quantity of RNA was assessed using a high sensitivity Tapestation (Agilent) to determine the RNA Integrity Number (RIN). All samples had a RIN value within the range of 8.0–9.5.

RNA libraries were prepared using a NEBNext® Ultra™ RNA library prep kit for Illumina® using TruSeq indexes. Libraries were prepared according to the manufacturer’s protocols. The final libraries were pooled and sequenced on a NextSeq 500 instrument (Illumina) using a paired-end run 2 × 41bp, to a minimum depth of 20 million paired-end reads/sample.

### RNA Sequencing Analysis

Reads sequenced following RNA-seq library preparation were mapped to the reference genome GRCh38 (hg38) using hisat v0.1.6 (22). Mapped reads were then assigned to an individual gene using featureCounts v1.4.6 (23), which is part of the SubReads package. Only uniquely mapped reads were used to assign reads to a gene. The resulting count tables were then used for differential gene expression analysis, which was performed using DESeq2 v1.12.3 within the R statistical framework v4.0.2.

### ChIP-Seq and Library Preparation

Chromatin immunoprecipitation (ChIP) was performed as previously described (20). All ChIPs were performed using single donors in triplicate. NK cells were cross-linked with formaldehyde, and chromatin was fragmented to 200 to 300 bp, using a Biorupter Pico sonicator (Diagenode). Immunoprecipitation was performed using 10 μg of anti-BRD2 (A302-583A, Bethyl Laboratories) or anti-BRD4 (A301-985A100, Bethyl Laboratories) antibodies. The ChIP experiment was then performed for each antibody as described previously by Orlando et al. (24). Next, library preparation was performed using a NEBNext Ultra DNA sample preparation kit (NEB), according to the manufacturer’s recommendations. The samples were then sequenced using a NextSeq 500 (Illumina) (single-end, 82 bp). Sequencing depth was >20 million reads per sample.

### ChIP Sequencing Analysis

In order to analyze the ChIP-seq datasets, a computational pipeline was generated using scripts from the CGAT toolkit and CGAT core workflow manager (25, 26). Reads were mapped to the GRCh38 assembly (hg38) using bowtie v0.12.5 (27). Bedtools version 2.2.24 was used to generate bedgraph files from mapped BAM files, and bigwig files were created using bedGrapohToBigWig (28). MACS2 software v2.2.7.1 was used to define peak enrichments of intervals of BRD2 and BRD4. Sequencing of the whole cell extract (input) was performed to determine the background model when analyzing ChIP-seq.

### Single-Cell Library Preparation

Single-cell libraries were generated using the drop-seq protocol (29). Briefly, cells were loaded into a Nadia (Dolomite Bio) microfluidics cartridge at a concentration of 310 cells per microliter. Cells were lysed in droplet, and RNA was captured onto Toyopearl oligonucleotide beads (ChemGenes). Emulsion formed from the microfluidics device was then isolated and droplets broken with 1H,1H,2H,2H-Perfluoro-1-octanol. Reverse transcription (RT) was then performed. Prior to PCR, the beads were treated with Exo-I, and then purified cDNA was used as an input for Nextera tagmentation reactions. cDNA library was assessed for quality using a TapeStation (Agilent Technologies) before being sequenced on a NextSeq 500 sequencer using a 75 base-pair cycle High Output kit (Illumina).

### Single-Cell RNA Sequencing Analysis

Raw sequencing data (FASTQ files) were processed using a CGAT-core computational pipeline, which is available at https://github.com/Acribbs/scflow. Reads were demultiplexed and then aligned to the GRCh38 (hg38) assembly reference genome using Salmon alevin v1.3.0 (30). Downstream processing of the cell by gene matrix was performed using Seurat v3.0 (31). We removed cells that expressed less than 300 genes, which resulted in the detection of 10,804 total cells.

Full description of the clustering performed is described in the Seurat documentation. Briefly, following quality control and filtering, gene expression for each cell was normalized and transformed. Highly variable genes that account for cellular heterogeneity were identified and cells were aligned using Harmony (32), then reduced using Uniform Manifold Approximation and Projection (UMAP) for dimension reduction. K562 cluster identification was performed based on the lack of MHC gene expression and K562 marker genes, which were identified from the Harmonizome database (33).

### Statistical Analyses

All values are presented as means ± S.D. Mann–Whitney U test and Kruskal–Wallis with Dunn’s test were used for multiple comparisons. Wilcoxon matched-pairs test was used for paired analyses. All calculations were performed using R or GraphPad prism software.

### Data and Software Availability

RNA-seq, ChIP-seq and single-cell RNA-seq datasets are deposited within GEO under the accession number GSE156423. A computational pipeline was generated using the CGAT-core workflow manager and the CGAT toolkit (25, 26). ChIP-seq pipelines are fully accessible on github https://github.com/cgat-developers/cgat-flow. Bulk RNA-seq R scripts are accessible *via* https://github.com/Acribbs/deseq2_report, while the single-cell RNA-seq pipelines can be accessed *via* https://github.com/Acribbs/scflow.

## Results

### BET Bromodomain Targeting Inhibits NK Cell Mediated Inflammatory Function

**Figure 2:**
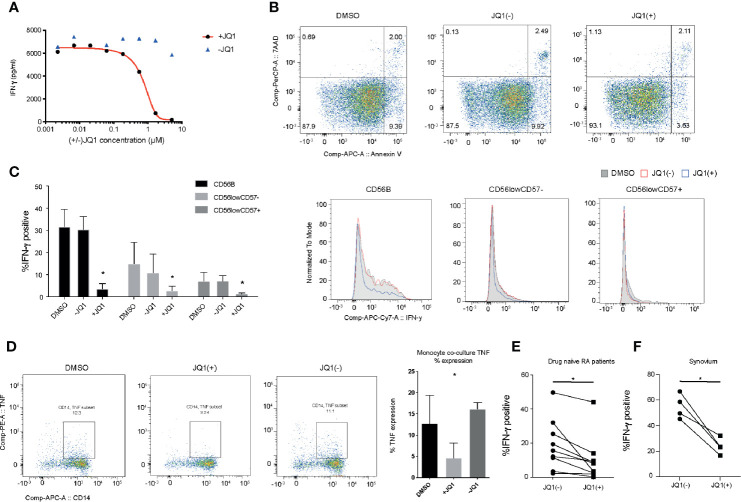
JQ1(+) bromodomain inhibitor inhibits NK cell mediated inflammatory function. **(A)** The measurement of IFN-**γ** in the culture supernatant following stimulation of NK cells with IL-15 (10 ng/ml) and 24 h of treatment with JQ1(+) and the negative control JQ1(−). **(B)** Flow cytometry analysis of 7-AAD and Annexin-V expression as markers for cell death in NK cells, following 48 h of treatment with DMSO, JQ1(+) or JQ1(−). **(C)** Flow cytometry analysis of IFN-**γ** staining for NK cells stimulated with IL-15 (10 ng/ml) and treated for 24 h with DMSO, JQ1(+) or JQ1(−). The frequency of IFN-**γ** positive cells is shown for CD56^bright^CD57^−^, CD56^dim^CD57^−^ and CD56^dim^CD57^+^ NK cell populations. **(D)** Flow cytometry analysis of CD14^+^ monocytes following co-culture with NK cells stimulated with IL-15 (10 ng/mL) and pre-treated with DMSO, JQ1(+) or JQ1(−) for 24 h. Cells were stained with CD14 and TNF-α and then analyzed by flow cytometry. **(E)** The frequency of IFN-**γ** positive cells in NK cells isolated from the peripheral blood of drug-naïve RA patients. **(F)** The frequency of IFN-**γ** positive cells in total NK cell populations isolated from RA synovial tissue. For **(E, F)**, P values were calculated using a Wilcoxon matched-pairs test. For **(C, D)**, P values were calculated using a Mann–Whitney U test. *P < 0.05. Error bars show mean ± SD (n = 3).

The hallmark of an NK cell is its ability to rapidly secrete a number of pro-inflammatory cytokines in response to activation. In a previous small molecule epigenetic compound screen, we identified a number of pan-BET bromodomain inhibitors, including JQ1(+) and PFi-1 that can reduce the production of pro-inflammatory IFN-**γ** cytokine (20). However, the molecular mechanism underpinning IFN-**γ** inhibition has only been partly investigated (18, 34). We confirmed the findings from our original screen by determining the IC50 for JQ1(+), the most potent BET bromodomain in our screen, in IL-15 stimulated primary human NK cells, derived from the peripheral blood of healthy individuals (**Figure 2A**). JQ1(+) is a BET bromodomain inhibitor that binds to the amino-terminal twin bromodomains of the BET family and shows excellent selectivity over other BRD-containing human proteins (12). We also included an inactive isomer, JQ1(−) as a negative control, demonstrating BET domain on-target effects of JQ1(+). Since reduced NK cell inflammatory function can also be ascribed to increased cell death, we investigated the cytotoxic effect of JQ1(+) on NK cells using Annexin-V and 7-AAD staining as a marker of apoptosis and cell death, respectively. We observed no significant increase in cell death following 48 h of culture with JQ1(+) (**Figure 2B**). Similarly, we observed no significant difference in the proliferation of NK cells following JQ1(+) treatment as determined by Ki-67 expression (**Supplementary Figure 1A**). Next, we evaluated the effect of JQ1(+) on the inflammatory function of different NK cell populations. JQ1(+) treatment resulted in reduced IFN-**γ** production in all NK cell subsets (CD56bright, CD56dimCD57+ and CD56dimCD57−); however, its effect was greatest on the CD56bright subset, while DMSO and the negative control compound JQ1(−) had little effect (**Figure 2C** and **Supplementary Figure 1**). In addition to IFN-**γ**, we also observed a reduction in TNFα and GM-CSF expression following 48 h of JQ1(+) treatment (**Supplementary Figures 2B, C**). In addition to intrinsic TNF-α production, NK cells also induce the expression of TNF-α in CD14+ monocytes in a cell contact-dependent mechanism. Therefore, we next evaluated the ability of pre-treated NK cells to induce monocyte derived TNF-α in co-cultures. We observed a reduction in monocyte TNF-α following IL-15 (10 ng/ml) and JQ1(+) pre-treatment of NK cells (**Figure 2D** and **Supplementary Figure 3**), demonstrating that BET inhibition can impact on NK cell/monocyte crosstalk. We next evaluated the ability of JQ1(+) to inhibit inflammation in a chronic inflammatory disease setting of Rheumatoid Arthritis (RA). Similar to healthy donors, we observed a significant reduction in the expression of IFN-**γ** in NK cells from the peripheral blood of drug naïve RA patients (**Figure 2E**). The synovial fluid from the joints of affected RA patients is enriched for NK cells that secrete high levels of IFN-**γ** in response to IL-15 treatment (20). We found a significant reduction in the expression of IFN-**γ** following treatment with JQ1(+), when compared to the JQ1(−) control (**Figure 2F**). Taken together, the results suggest that the pan-BET bromodomain inhibitor JQ1(+) can reduce inflammatory function of NK cells in both an acute and chronic inflammatory situation.

### Knockdown of BRD2 and BRD4 Suppresses the Inflammatory Function of NK Cells

**Figure 3:**
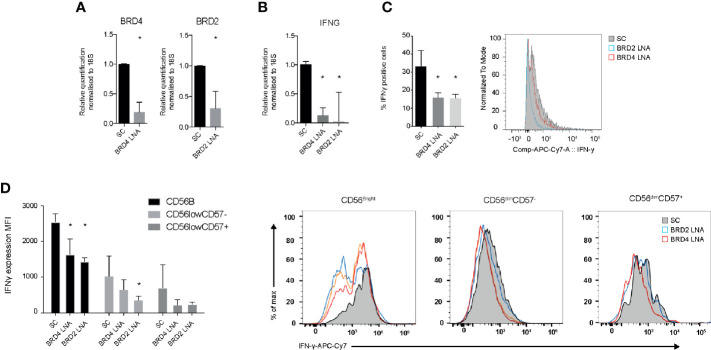
BRD2 and BRD4 regulate NK cell inflammatory function. NK cells were cultured in the presence of IL-15 and either scrambled control (SC), BRD2 or BRD4 LNA oligonucleotide (n = 5). **(A)** Knockdown efficiency was assessed using qPCR. **(B)** The expression of IFNG gene expression was measured using qPCR. **(C)** The expression of IFN-**γ** protein in total NK cells was measured using flow cytometry. **(D)** NK cells were further gated based on the expression of CD56 and CD57 and then intracellular IFN-**γ** was measured. A representative figure shows the median fluorescent intensity of IFN-**γ** among CD56^bright^CD57^−^, CD56^dim^CD57^−^ and CD56^dim^CD57^+^ NK cell populations. The right panel shows a representative flow cytometry histogram of IFN-**γ** expression in each NK cell subpopulation. The data are from three independent experiments. The data represents means ± S.D. in **(A–D)**. P values were calculated using Kruskal–Wallis with Dunn’s multiple comparison test *P < 0.05. MFI, median fluorescence intensity.

To evaluate the specificity of JQ1(+) compound on NK cell function, we used locked nucleic acid (LNA) knockdown of BRD2 and BRD4, the previously validated targets of JQ1(+). We show efficient knockdown of both BRD2 and BRD4 when compared to a scrambled control LNA, as measured using Rt-PCR (**Figure 3A**). Both BRD2 and BRD4 were found to significantly reduce the expression of IFN-**γ** mRNA following IL-15 stimulation (**Figure 3B**). Next we measured the expression of IFN-**γ** protein expression by flow cytometry. These data confirm a significant reduction in the IFN-**γ** protein expression following BRD2 and BRD4 knockdown (**Figure 3C**). The reduction in IFN-**γ** was apparent in all NK cell subsets (CD56bright, CD56dimCD57+ and CD56dimCD57−), with the knockdown effect being greatest in the CD56bright NK cell population, which was an expected observation (**Figure 3D**). Taken together, these data demonstrate that both BRD2 and BRD4 BET proteins are key regulators of the inflammatory response within NK cell populations.

### BET Targeting in NK Cells Imparts an Anti-Inflammatory Phenotype and a Reduction in the Expression of Cytolytic Markers

**Figure 4:**
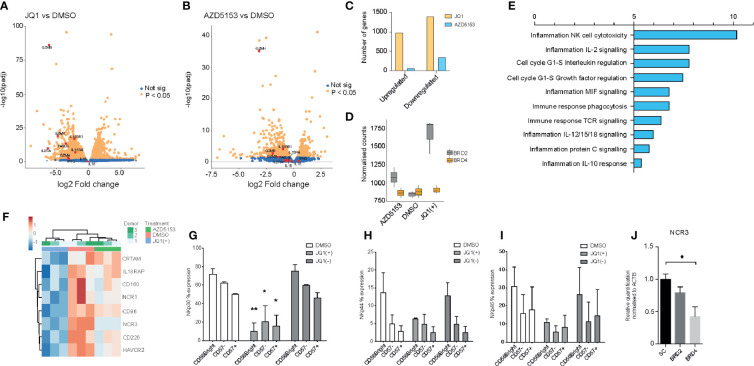
JQ1(+) and AZD5153 impact on NK cell inflammatory function. NK cells were cultured in the presence of IL-15 (10 ng/ml) and treated with either DMSO, JQ1(+) or JQ1(−) for 24 h. **(A)** A volcano plot showing the gene expression differences between JQ1(+) and DMSO treated NK cells. **(B)** A volcano plot showing the gene expression differences between AZD5153 and DMSO treated NK cells. **(C)** A Bar chart showing the number of upregulated and downregulated genes following JQ1(+) or AZD5153 treated NK cells. **(D)** Box plots showing the normalized counts of BRD2 and BRD4 following treatment with DMSO, JQ1(+) or AZD5153. **(E)** Pathway enrichment analysis showing the metacore process pathways for the differences in gene expression between JQ1(+) and AZD5153 treated NK cells. **(F)** Heatmap of gene expression changes in three individual NK cell donors upon treatment with either DMSO, JQ1(+) or JQ1(–). The percent expression of NKp30 **(G)**, NKp44 **(H)** and NKp46 **(I)** as measured by flow cytometry. **(J)** The relative quantification of NCR3 following culture with scrambled control (SC), BRD2 or BRD4 LNA, as measured by qPCR. Data in **(G–I)** represents mean ± S.D. and is measured in three independent donors. P values were calculated using Kruskal–Wallis with Dunn’s multiple comparison test *P < 0.05, **P < 0.01.

The observed anti-inflammatory phenotype following treatment with JQ1(+) prompted us to investigate the global transcriptional changes in NK cells following BET bromodomain inhibition. Given the non-selective property of JQ1(+) between BET bromodomain members, we included the small molecule inhibitor AZD5153 to specifically delineate the effect of BET bromodomain inhibition in NK cells. Unlike the JQ1(+) monovalent BET inhibitor, AZD5153 interacts with both bromodomains simultaneously and has greater specificity for BRD2 than JQ1(+), which has a preferential affinity for BRD4 (11, 15) (**Figure 1**). We determined EC50 values for AZD5153 by measuring the reduction of IFN-**γ** in culture supernatant and found it has 30-fold greater potency than JQ1(+), with an EC50 of 0.02 µM (**Supplementary Figure 4A**). Transcriptomic experiments were performed by bulk RNA sequencing (RNA-seq) in IL-15 stimulated NK cells following 24 h of treatment with JQ1(+), AZD5153 or DMSO control. Using stringency criteria of a +/− 2 log two-fold increase and false discovery rate of 0.05, this revealed 449 downregulated genes and 432 upregulated genes following JQ1(+) treatment (**Figure 4A**), while AZD5153 treatment resulted in 102 downregulated genes and nine upregulated genes following AZD5153 treatment (**Figure 4B**). We observed a higher number of differentially regulated genes following JQ1(+) treatment, when compared to AZD5153 (**Figure 4C**). Interestingly, we observed a stark and significant increase in the expression of BRD2 but not BRD4 following treatment of NK cell cultures with both BET bromodomain inhibitors, with the effect being more pronounced with JQ1(+) (**Figure 4D**). This suggests a possible regulatory feedback mechanism for controlling BRD2 expression in NK cells. To identify common NK cell specific BET bromodomain targets, we performed pathway analysis on overlapping differentially regulated genes between JQ1 and AZD5153 treated NK cells. No significant differences in pathways were detected, but we did identify three transcription factors IKZF4, BATF, and BATF3 as being differentially regulated by both JQ1(+) and AZD5153 (**Supplementary Figure 4B**). Next we compared the non-overlapping gene expression changes between JQ1(+) and AZD5153 and identified a significant enrichment of genes downregulated for processes that include NK cell cytotoxicity, IL-2 signaling and cell-cycle response (**Figure 4E**). When we further investigated the genes associated with differences in the NK cell cytotoxicity process, we identified NCR1 and NCR3 as being significantly downregulated with JQ1(+), but not with AZD5153 (**Figure 4F**). Given that NCR1 and NCR3 genes encode for the NK cell activation receptors and are important for priming NK cells to induce cytolytic function, we next investigated the expression of these key receptors at the protein level by flow cytometry. We found significant reductions in the expression of NKp30 (NCR3) (**Figure 4G**) and a trend towards reduced expression of NKp44 (NCR2) (**Figure 4H**) and NKp46 (NCR1) (**Figure 4I**) following JQ1(+) treatment. Knockdown of BRD4 led to a reduction in NKp30 expression, while no reduction was observed following BRD2 knockdown (**Figure 4J**).

### Identification of Commonly Regulated Gene Expression Signatures Across Major Inflammatory Cell Populations

**Figure 5:**
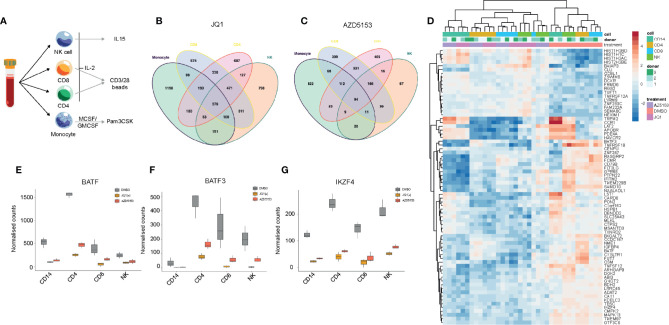
BET bromodomain inhibitors have a broad anti-inflammatory effect on the immune system. **(A)** A schematic showing the experimental setup. NK, CD8, CD4 and monocytes were isolated from healthy peripheral blood and then cultured in the presence of cytokines for 24 h before being stimulated with either IL-15 (10 ng/ml), CD3/28 activation beads (1:1 ratio) or MCSF/GMCSF (10 ng/ml). **(B)** A Venn diagram showing the overlapping differentially regulated genes for NK, CD4, CD8 and monocytes following 24 h of JQ1(+) treatment. **(C)** A Venn diagram showing the overlapping differentially regulated genes for each NK, CD4, CD8 and monocytes following 24 h of AZD5153 treatment. **(D)** A heatmap showing the common genes regulated between JQ1(+) and AZD5153 across all inflammatory cells. **(E**–**G)** Box plots showing the normalized counts of BATF, BATF3 and IKZF4 transcription factors in NK, CD4, CD8 and monocytes following JQ1(+) or AZD5153.

BET bromodomain inhibition has been shown to elicit a general anti-inflammatory effect in many immune cell types (35). We next wanted to utilize this general anti-inflammatory property of BET bromodomain inhibitors to assess common gene regulatory changes across all inflammatory cells to identify key transcription factors. We isolated CD4+, CD8+, monocytes, and NK cells from peripheral blood of healthy volunteers. These cells were cultured with either IL-2 or GMCSF/MCSF cytokines for 24 h before NK cells; CD4/CD8 cells or monocytes were stimulated with either IL-15, CD3/28, or Pam3CSK, respectively (**Figure 5A**). RNA-seq was then performed on cells cultured following 24 h of DMSO, JQ1(+), or AZD5153 treatment. In line with our NK cell data, we found that JQ1(+) regulated more genes than AZD5153 across all inflammatory cells (**Figures 5B, C**). Common gene expression signatures were identified between JQ1(+) and AZD5153 cultured inflammatory cells (**Figure 5D**). Interestingly, BATF (**Figure 5E**), BATF3 (**Figure 5F**), and IKZF4 (**Figure 5G**) transcription factors were downregulated following both JQ1(+) and AZD5153 treatment across all inflammatory cells. Overall, the fact that there were very few regulated transcription factors suggests that BET bromodomains may mediate their functions, in part, through a controlled BATF and IKZF4 response.

### NK Cell Treatment With JQ1(+) but not AZD5153 Results in Compromised NK Cell Mediated Cytotoxicity

**Figure 6:**
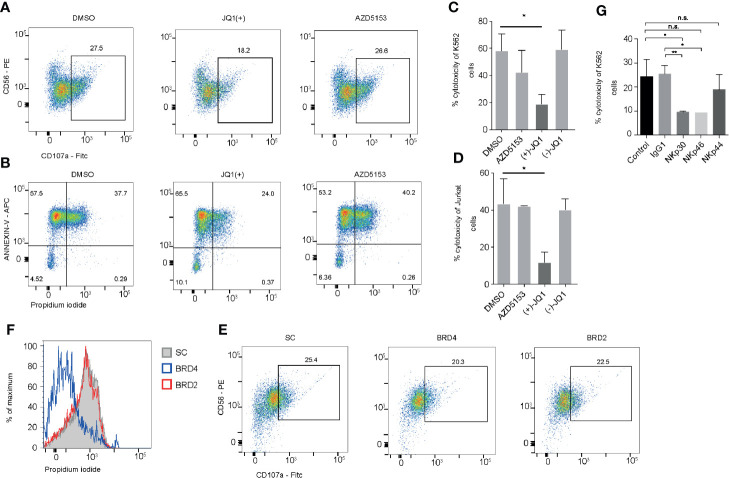
BRD2 inhibition impairs NK cell killing function. **(A)** NK cells were stimulated with IL-15 (10 ng/ml) and then treated with DMSO, JQ1(+) or AZD5153 for 24 h. NK cells were then co-cultured with K562 cells for 4 h and CD107a was measured by flow cytometry **(B)**. Flow cytometry was also used to measure cell death in CD56^-^ K562 cells by measuring the expression of Annexin-V and propidium iodide uptake. **(C)** An LDH release assay was used to measure NK cell mediated cell lysis of K562 cells following 24 h of treatment with DMSO, JQ1(−), JQ1(+) or AZD5153. **(D)** An LDH release assay showing the % cytotoxicity of Jurkat cells following 24 h of NK cell treatment with DMSO, JQ1(−), JQ1(+) or AZD5153. **(E)** NK cells were cultured in the presence of scrambled control (SC), BRD2 or BRD4 LNA and then CD107a expression was measured following co-culture with K562 cells. **(F)** The level of killing of K562 cells was determined by measuring propidium iodide uptake. **(G)** Blocking antibodies against IgG1, NKp30, NKp44, NKp46 were added to NK cells stimulated with IL-15 (10 ng/ml). An LDH release assay was then performed and the % cytotoxicity of K562 cells was measured using an LDH release assay. **(A, B, E, F)** are representative of three independent experiments. **(C, D, G)** show mean ± S.D. of three independent experiments. P values were calculated using Kruskal–Wallis with Dunn’s multiple comparison test *P < 0.05, **P < 0.01. n.s., not significant.

Having identified that JQ1(+) but not AZD5153 treatment inhibits a number of key NK cell mediated cytotoxicity genes, we next investigated whether this could impact on the cytolytic function of NK cells. NK cells were pre-treated with either DMSO control or BET inhibitors JQ1(+) or AZD5153 for 24 h and then washed twice before measuring cytolytic activity using CD107a surface expression and cell death of K562 target cells by Annexin-V and Propidium iodide uptake (21). This revealed a significant reduction in CD107a expression on NK cells and a reduction in the ability of NK cells to kill target cells following JQ1(+) but not AZD5153 or DMSO control (**Figures 6A, B**). We confirmed these results using an LDH release assay against both K562 target cells (**Figure 6C**) and Jurkat cells (**Figure 6D**). Given that compromised NK cell killing was only observed with JQ1(+) and not AZD5153, we reasoned that this could be due to the differences in the affinity they have for the different BET bromodomain family members (**Figure 2**). Therefore, we next investigated the role of BRD2 and BRD4 in regulating NK cell killing function using locked nucleic acid knockdown experiments. Only following BRD4 but not BRD2 knockdown we observed compromised NK cell killing function (**Figure 6E**). However, there was only a small reduction in CD107a following either BRD4 or BRD2 knockdown (**Figure 6F**). To evaluate the relative importance of each NK cell activating receptor on NK cell killing, we next performed blocking experiments. We found that blocking NKp30 (NCR3) and NKp46 (NCR1) led to a reduction in the ability of NK cells to kill their target cells, while NKp44 (NCR2) blocking had little effect on NK cell mediated killing (**Figure 6G**). Taken together, these data suggest that both BRD2 and BRD4 can regulate the inflammatory function of NK cells, but only BRD4 expression is critical for normal NK cell cytolytic activity, explained in part by the reduction in the expression of NKp30.

### Single-Cell Sequencing Reveals a Heterogeneous Response to BET Bromodomain Inhibition in NK Cells

**Figure 7:**
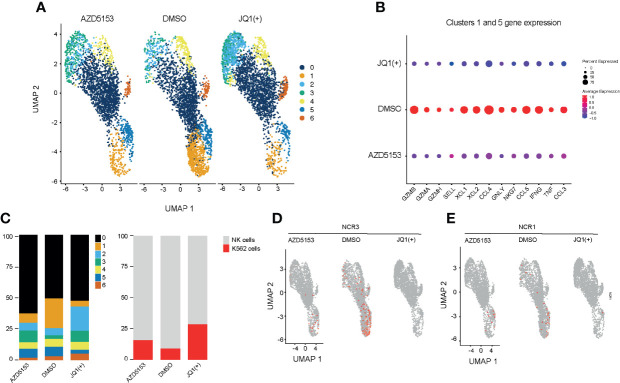
Single-cell transcriptomics reveals a heterogeneous response to BET bromodomain inhibition. NK cells were stimulated with IL-15 (10ng/mL) and treated with DMSO, JQ1(+) or AZD5153 for 24 h. NK cells were then co-cultured with K562 cells for 2 h and single-cell RNA sequencing was performed. **(A)** A Uniform Manifold Approximation and Projection (UMAP) plot showing clustering and differences between DMSO, JQ1(+) and AZD5153 treated NK cells in co-culture with K562 cells **(B)** The average gene expression in highly inflammatory NK clusters 1 and 5 between DMSO, JQ1(+) and AZD5153 treated NK cells. **(C)** Bar charts showing the relative proportion for each cluster in all treatment groups. The right bar chart shows the relative cell proportions of NK cells to K562 cells. **(D)** UMAP plots showing the expression of NCR3 between DMSO, JQ1(+) and AZD5153 treated NK cells. **(E)** UMAP plots showing the expression of NCR1 between DMSO, JQ1(+), and AZD5153 treated NK cells.

Next we investigated the effect of BET bromodomain inhibitors on cytolytic killing using single-cell killing assay. To examine the transcriptional response to BET inhibitors in single-cells, we pre-treated NK cells with either DMSO control, JQ1(+), or AZD5153 for 24 h and then co-cultured them in the presence of K562 target cells for 4 h. We performed droplet-based single-cell sequencing (11,970 total cells) across all three conditions (29). We then filtered out cells that expressed less than 300 genes and performed dimensionality reduction by unsupervised clustering of the 10,804 recovered cells using the Seurat R package (31). Given our interest in measuring K562 cell death, we did not remove cells with high levels of mtRNA. To align our datasets and remove potential batch effects we used Harmony (32) and visualized clustering following Uniform Manifold Approximation and Projection (UMAP) dimensional reduction (36) (**Figure 7A**). We identified seven clusters in total based on the transcriptional profile of cells, two representing K526 cells (Clusters 2 and 3) and five clusters of NK cells. K562 cells were identified based on the lack of MHC expression and high expression of K562 marker genes, selected from the Harmonizome database (33). Clusters 1 and 5 express high levels of inflammatory cytokines and cytolytic genes, which were significantly reduced with both JQ1(+) and AZD5153 pre-treatment (**Figure 7B** and **Supplementary Figure 5**). Additionally, we also observed a clear reduction in the composition of these clusters, and in cell numbers following both JQ1(+) and AZD5153 treatment.

We next compared the relative proportion of K562 to NK cell clusters to determine the level of NK cell mediated cytolytic killing (**Figure 7C**). In line with our killing experiments we identified a greater number of K562 cells in JQ1(+) pre-treated NK cells (27%) when compared to both AZD5153 (15%) and DMSO control (8%). Given that our previous flow cytometry experiments showed a clear reduction in the expression of NK cell activation markers following JQ1(+) treatment, we next evaluated the expression of NCR1 and NCR3. Despite generally low expression levels for these transcripts, the expression of both NCR1 and NCR3 was absent within the JQ1(+) pre-treated NK cells and thus confirms our previous bulk RNA-seq results (**Figures 7D, E**). Although the expression of both genes was reduced in AZD5153 treated cells, a proportion of cells still expressed both NCR1 and NCR3. Overall, these results confirm our previous bulk RNA-seq and NK cell killing experiments showing that there is a significantly attenuated cytolytic response when NK cells are treated with JQ1(+), but the effect on cytolytic function is not as apparent when cultured with AZD5153.

### BET Bromodomain Inhibition Reduces Both BRD2 and BRD4 Binding to Promoters

**Figure 8:**
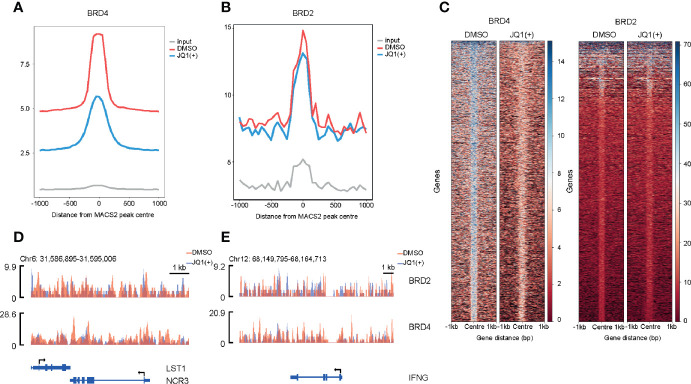
Genome-wide BRD2 and BRD4 occupancy following stimulation of NK cells with IL-15 (10 ng/ml) and 24 h of JQ1(+) treatment of NK cells. **(A)** Coverage plot showing average read counts centered around the transcriptional start site (TSS) from BRD4 ChIP-seq following DMSO or JQ1(+) treatment of NK cells. **(B)** Coverage plot showing average read counts centered around the TSS from BRD2 ChIP-seq following DMSO or JQ1(+) treatment of NK cells. **(C)** Heatmap of BRD4 and BRD2 following treatment of NK cells with DMSO or JQ1(+) covering 1 kb upstream and downstream of the center of called peaks. **(D)** Representative ChIP-seq coverage profiles for BRD2 and BRD4 across the *NCR3* locus. **(E)** Representative ChIP-seq coverage profiles for BRD2 and BRD4 across the *IFNG* locus.

In order to understand the mechanisms of how BRD4 and BRD2 regulate NK cell inflammatory and cytolytic function, we performed genome-wide occupancy studies using chromatin immunoprecipitation for BRD4 and BRD2, followed by sequencing. These studies revealed a significantly reduced occupancy of BRD4 and a modest but distinct reduction in BRD2 occupancy throughout the genome following. JQ1(+) treatment (**Figures 8A, B**). The reduction of BRD4 following JQ1(+) treatment is most striking when compared to BRD2 (**Figure 8C**). However, both NCR3 and IFNG show a marked decrease in both BRD4 and BRD2 occupancy following treatment with JQ1(+) (**Figures 8D, E**). Altogether, these results are consistent with a positive role of gene transcription for both BRD4 and BRD2 within NK cells. Moreover, the profile of BRD2 occupancy is more restricted than that of BRD4, suggesting that BRD2 may regulate fewer genes than that of BRD4.

## Discussion

Our work provides novel insight into the epigenetic regulation of NK cells within an inflammation and cancer context. A handful of studies identified DNA methylation (37), histone acetylation (38), EZH2, and SUZ12 methyltransferases (39), polycomb repressor complex 2 (PRC2) and jumonji histone demethylases as playing a significant role in the inflammatory function of NK cells (20). Similarly, BET bromodomains have also been implicated in NK cell function, with selective inhibition of BET bromodomains using JQ1(+) being shown to repress MYC mediated super-enhancer activity and suppress IFN-**γ** expression in NK and Th1 polarized cells (18). Furthermore, JQ1(+) was shown not to alter H3K27ac or H3K27me3 chromatin marks, but was found to displace RNA polymerase II from the locus (18). Furthermore, IFN-**γ** repression was found to be reversible, since the removal of JQ1(+) led to the recovery of Th1 cells to express IFN-**γ**; however this was not demonstrated for NK cells. The anti-inflammatory properties of JQ1(+) are fully confirmed by our current study. Furthermore, we show that JQ1(+) has wider anti-inflammatory effects, as it also impacts on the ability of NK cells to induce a pro-inflammatory response in monocyte co-cultures. The anti-inflammatory effects were found to be due to both BRD4 and BRD2 since knockdown of these two BET bromodomains led to reduced NK cell inflammatory function.

We were also able to show that a second BET bromodomain inhibitor, AZD5153 also imparts an anti-inflammatory effect on NK cells. Our single-cell experiments were able to show a significant response of specific NK cell subsets to both JQ1(+) and AZD5153 treatment. In these experiments, highly inflammatory NK cells show the highest level of gene transcriptional changes, with a significant reduction in a broad range of inflammatory genes. Our bulk RNA-seq data revealed that JQ1(+) treatment led to a downregulated NK cell cytolytic response, which impacted NK cell killing of cancer cells, in part through the downregulation of NK cell activating receptors. Consistent with these findings, JQ1 has been shown to downregulate target ligands in neuroblastoma cells and downregulate NK cell activation receptors NKG2D and DNAM-1 on NK cells (40). Unlike inflammatory function, where both BRD2 and BRD4 were found to play a role in the regulation of cytokine genes, we show that when only BRD4 is perturbed do we see a significant reduction in the ability of NK cells to eliminate cancer target cells. Furthermore, the significant downregulation of NCR3 (NKp30) in both the bulk and single-cell transcriptomic data, combined with our blocking experiments, suggests that compromised NK cell function is mediated, in part, by the reduction in NKp30 (NCR3). Interestingly, the impact on NK cell cytolytic function was only observed with JQ1(+) treatment and not AZD5153, which may be explained by the different affinity or different bio-availability each compound has for the different BET bromodomain family members.

JQ1(+) is a monovalent BET inhibitor that does not engage simultaneously the two bromodomains found in the BET family members. It has been shown to be more effective in hematopoietic cancers, but is far less effective in other solid tumors, such as breast cancer and cervical cancers (11, 12, 41–46). Consequently, this has spurred the development of bivalent bromodomain inhibitors, engaging with both bromodomains, such as AZD5153 (15), which has higher affinity for BRD2 and lower affinity for BRD4 when compared to JQ1(+) (11). The different affinities for BRD2 and BRD4 between JQ1(+) and AZD5153 may help explain the different cytolytic inhibitory effects of the two compounds. Indeed, our knockdown assays demonstrate that NK cell mediated cytotoxicity is regulated by BRD4 activity and not by BRD2. This suggests that BRD4 plays a significant role in the regulation of NK cell cytolytic function, which seems to be independent of BRD2 function. Despite this, it is clear that both BRD2 and BRD4 are important for mediating NK cell inflammatory function, since knockdown of both gave a clear reduction in the expression of IFN-**γ** and TNF-α. Interestingly, we also observed a reduction in several other pro-inflammatory cytokines including, IL-12 and GM-CSF but also for anti-inflammatory IL-10. This suggests that BET bromodomains regulate the expression of both pro- and anti-inflammatory cytokine production within NK cells. Reduced IL-10 expression following BET bromodomain inhibition is consistent with findings within B cells (47), but is at odds with findings in CD4^+^ T cells, where IL-10 is increased following BET bromodomain inhibition (48). This likely points to a more complex regulation of IL-10 that likely depends on cell type specific transcription factor expression.

Much more is known about the regulation of gene expression by BRD4 than BRD2 and very little is known about both of their functions in NK cell biology. BRD4 recruits PTEF-b to sites of active transcription, which in turn phosphorylates RNA polymerase II Ser2 during elongation (49). Thus, BRD4 regulates the conversion of basal transcription to higher rates of active elongation directly through RNA polymerase II (50). Furthermore, BRD4 can independently recruit transcriptional activators, such as NSD3, JMJD6 and CHD4 to remodel chromatin (51). Less is known about BRD2 transcriptional regulation, with its role being ascribed to act as a scaffold for the recruitment of histone acetyltransferases, histone deacetylases and E2F, thereby coupling histone acetylation to transcription in a PTEF-b-independent mechanism (52, 53). BRD2 enhances the passage of RNA polymerase II through acetylated chromatin within the gene body and is essential during development as Brd2−/− is embryonic lethal (54) while heterozygous mice are viable but have low numbers of neurons and develop spontaneous seizures (55). BRD2 is also important for mediating inflammation in murine macrophages (56). Our study adds important knowledge about BET bromodomain regulation of inflammation. We identify three common transcription factors, BATF, BATF3 and IKZF4 that are regulated across multiple inflammatory cells. Indeed, it has been shown that BRD2 interacts with the CTCF-cohesion complex and the STAT3–IRF4–BATF complex in Th17 cells (57). Our data points to the role of this complex as a general mechanism of BET bromodomain regulation across all inflammatory cells. The importance of BATF within the immune system has been well established, with BATF and BATF3 mainly involved in the immune regulation of dendritic cells, T cells and B cells, while BATF2 is mainly expressed in monocytes and Th1 cells (58–60). Moreover, BET bromodomains regulate Th17 differentiation using mechanisms that require BATF (61). Currently, little is known about the role of BET bromodomain regulation of IKZF4, but our data suggests that it may play a more generalized role in inflammatory cells. Although not investigated within this study, these findings warrant further work to explore their role within the wider context of inflammation. Likely this will lead to a better understanding of the transcriptional responses elicited by BET bromodomain inhibition. Currently there are at least 14 different small molecule inhibitors undergoing clinical trials for different forms of cancer, type 2 diabetes and cardiovascular disease (8, 35). We anticipate that our study will contribute to understanding the role and use of BET bromodomain inhibitors within the clinic. Specifically, given that our findings show that NK cell killing may be compromised following BRD4 but not BRD2 inhibition, this may help to explain any failure to meet study endpoints. Moreover, our results also provide a rationale for prioritizing BET bromodomain inhibitors that have a higher affinity for BRD2 than BRD4.

## Data Availability Statement

RNA-seq, ChIP-seq and single-cell RNA-seq datasets are deposited within GEO under the accession number GSE156423. A computational pipeline was generated using the CGAT-core workflow manager and the CGAT toolkit (25, 26). ChIP-seq pipelines are fully accessible on github https://github.com/cgat-developers/cgat-flow. Bulk RNA-seq R scripts are accessible via https://github.com/Acribbs/deseq2_report, while the single-cell RNA-seq pipelines can be accessed via https://github.com/Acribbs/scflow.

## Ethics Statement

The studies involving human participants were reviewed and approved by the Oxford Research Ethics Committee and the London Riverside Research Ethics Committee (REC numbers: 07/H0706/81 and 06/Q1606/139). The patients/participants provided their written informed consent to participate in this study.

## Author Contributions

AC, MF, and UO: conceptualization and designed research. AC, HO, VV-A, and UO contributed new reagents/analytical tools. AC, PF, MP, and GW: performed research. HP: provided patient samples and metadata. AC, PF, and UO analyzed data. AC and UO: writing-original manuscript draft. AC and UO: writing-review and editing. All authors contributed to the article and approved the submitted version.

## Funding

The study was supported through funding from the Kennedy Trust for Rheumatology Research, the National Institute for Health Research Oxford Biomedical Research Unit (UO, MF), Cancer Research UK (CRUK, UO), Arthritis Research UK (20522) (UO), the Bone Cancer Research Trust (BCRT) (AC and UO), and a Leducq Epigenetics of Atherosclerosis Network program grant from the Leducq Foundation (UO). UO was supported by the People Programme (Marie Curie Actions) of the European Union’s Seventh Framework Programme (FP7/2007–2013) under REA grant agreement n° [609305]. AC was supported by the Medical Research Council (MRC) CGAT program (G1000902), MRC Career Development Fellowship (MR/V010182/1) and a CRUK Oxford Centre Development Fund Award (CRUKDF-0318-AC[AZ]), with support from AstraZeneca. PF was supported by the Medical Research Council (MR/N010051/1).

## Conflict of Interest

VV-A was employed by AstraZeneca. HO was employed by Roche Innovation Center Copenhagen A/S.

The remaining authors declare that the research was conducted in the absence of any commercial or financial relationships that could be construed as a potential conflict of interest.​

The authors declare that this study received funding from AstraZeneca. The funder was involved in the interpretation of the data and reviewed the article before the decision to submit it for publication.
