# Supplementary material for: Dissecting the Role of BET Bromodomain Proteins BRD2 and BRD4 in Human NK Cell Function
Source: Front Immunol. 2021 Feb 26;12:626255. doi: 10.3389/fimmu.2021.626255 (PMC7953504; doi:10.3389/fimmu.2021.626255)
Supplement: Supplementary file 3 [file DataSheet_3.docx]

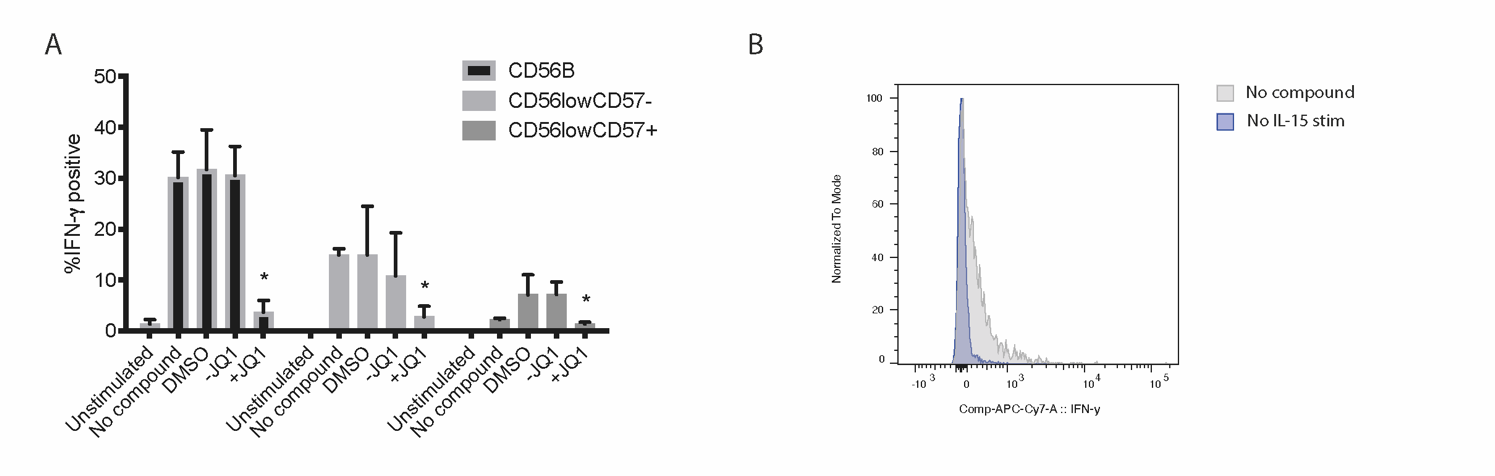
Supplementary Material

Figure 1. **A** A companion figure to Figure 2C showing the expression of IFN-γ for unstimulated NK cells and No compound treated NK cells. **B** Representative flow cytometry plot showing unstimulated and no compound controls in the CD56^bright^ NK cell population. P values were calculated using a Mann-Whitney U test. * P<0.05. Error bars show mean ± SD (n = 3)


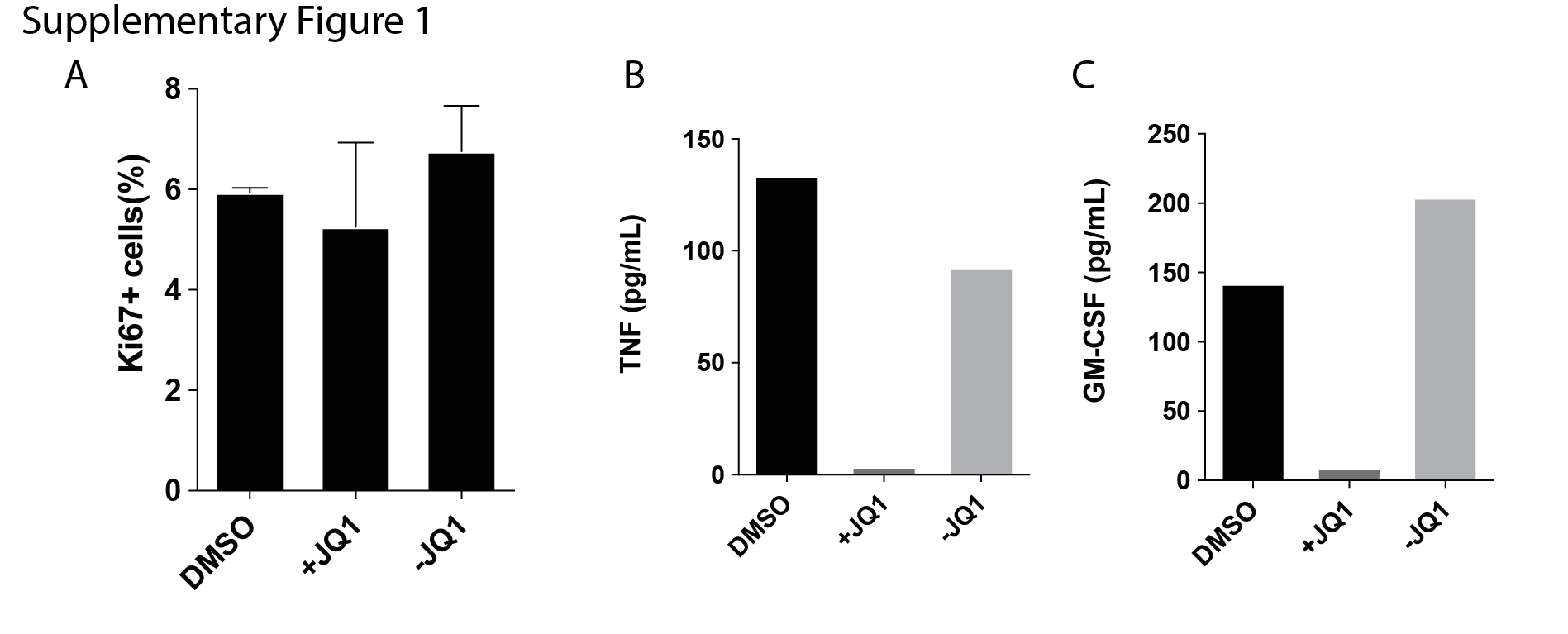


Figure *2*: NK cells treated with (+)JQ1 reduced NK cell inflammatory function. (A) NK cells treated with DMSO, (+)JQ1 or (-)JQ1 for 24 hours and then the expression of Ki67 was determined by flow cytometry. (B and C) NK cells treated with DMSO, (+)JQ1 or (-)JQ1 for 24 hours and then TNF (B) and GMCSF (C) was measured by ELISA.

*
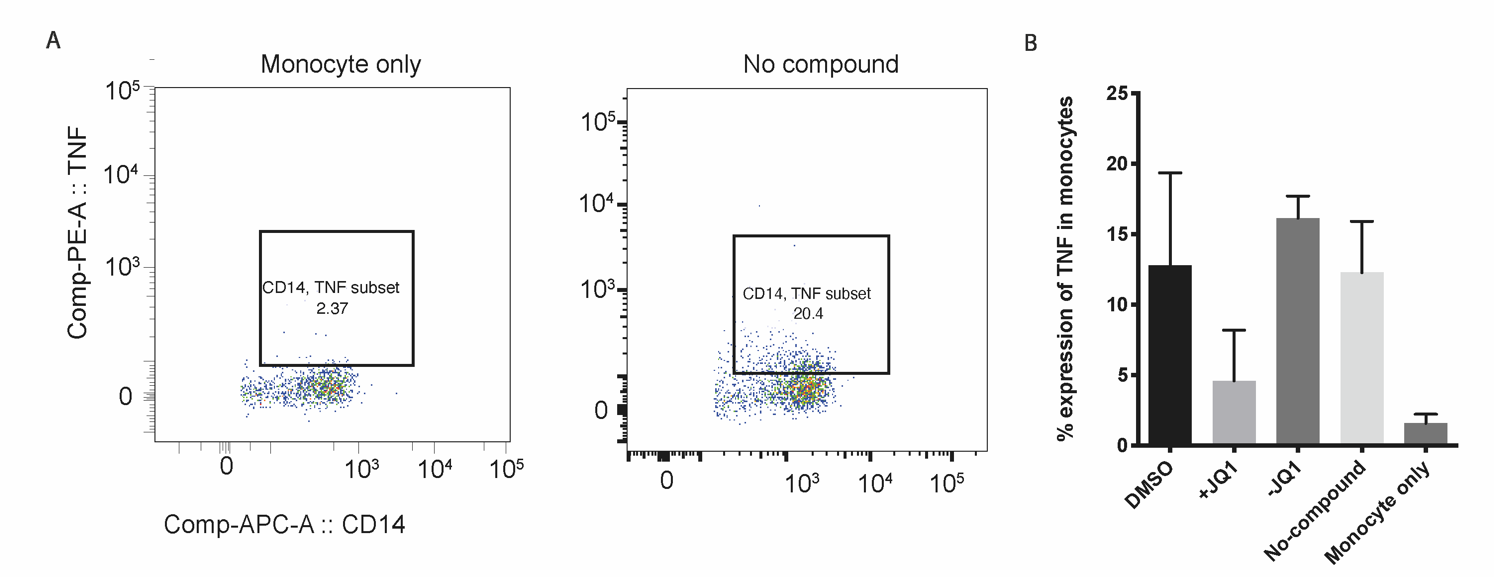
*

Figure 3: **A** Companion to figure 2D showing the expression of TNF in monocytes only (left panel) and NK cells that were stimulated with IL-15 (10ng/mL) without compound pre-treatment (right panel). **B** Bar plot showing the expression of TNF in CD14^+^ monocytes following co-culture with NK cells stimulated with IL-15 (10ng/mL) and pre-treated with DMSO, JQ1(+) or JQ1(-) for 24 hours.


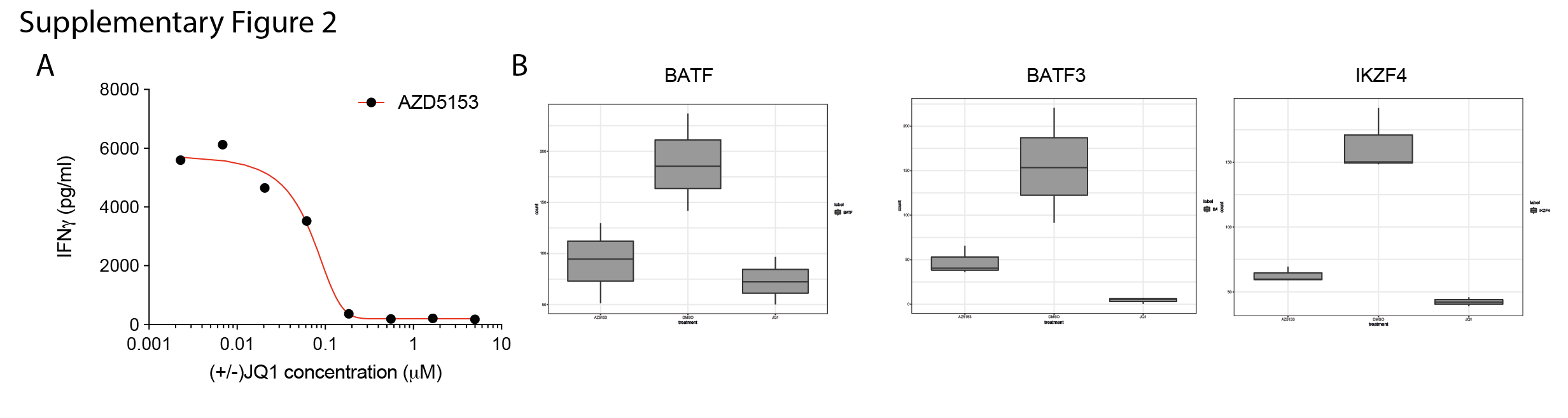


Figure *4*: (A) The measurement of IFN-γ in the culture supernatant following treatment with AZD5153. (B) NK cells were cultured in the presence of IL-15 and treated with either DMSO, JQ1(+) or JQ1(-) for 24 hours. Box plots showing the normalised counts of BATF, BATF3 and IKZF4 following treatment with DMSO, JQ1(+) or AZD5153.


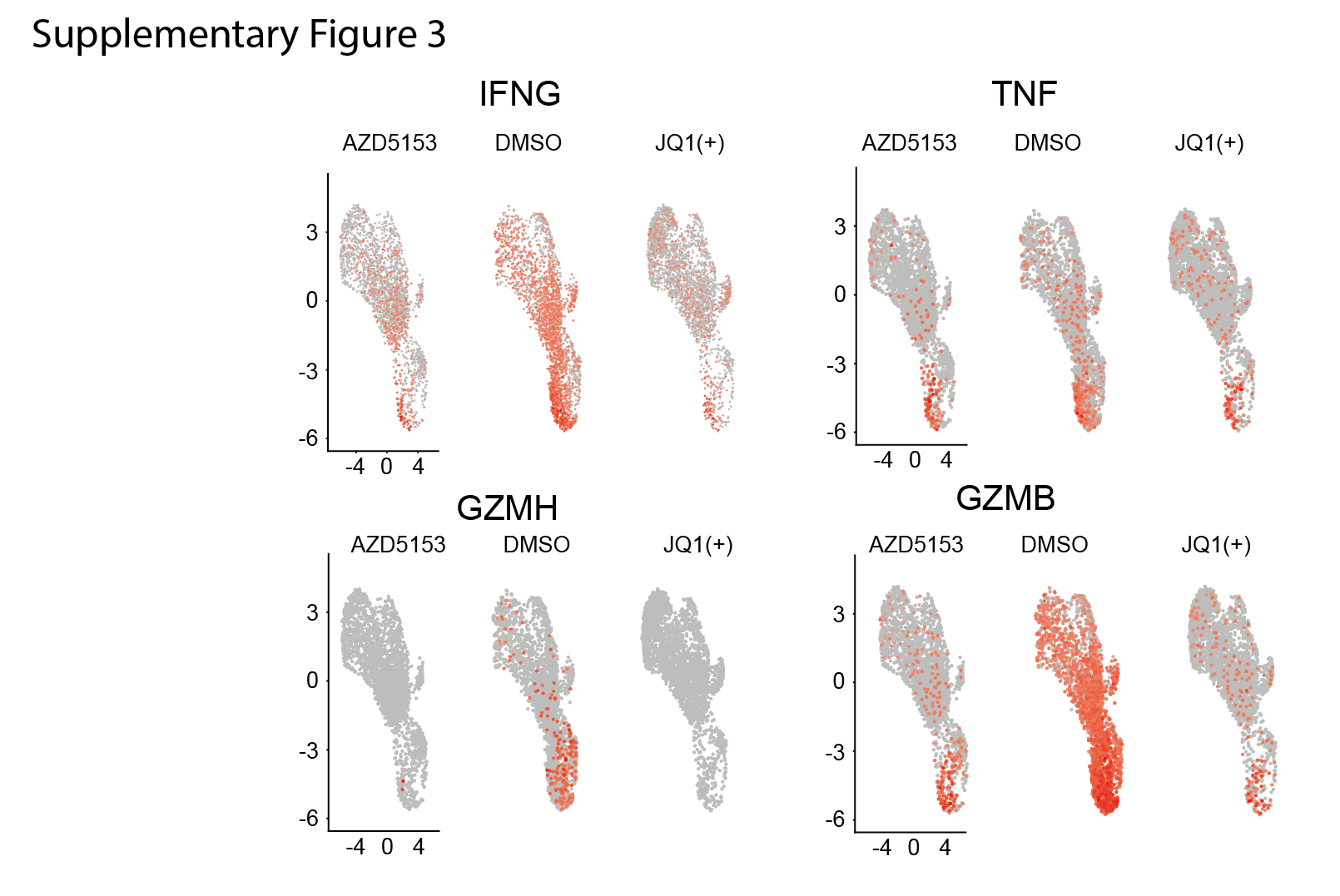


Figure *5*: UMAP plots showing the expression of IFNG, TNF, GZMH and GZMB between DMSO, JQ1(+) and AZD5153 treated NK cells.
